# Surgical treatment of pathological femoral neck fracture ending in push-through total femoral endoprosthesis. A case report

**DOI:** 10.3205/iprs000191

**Published:** 2025-08-22

**Authors:** Mohamed Ghanem, Christina Pempe, Andreas Roth

**Affiliations:** 1 Department of Orthopedics, Traumatology and Plastic Surgery, University Hospital Leipzig, Germany; 2 Department of Physical Therapy and Rehabilitation, University Hospital Leipzig, Germany

## Abstract

Arthroplasty in managing tumors of the extremities is a challenging surgery. Careful planning and the expertise of the surgical team are of utmost importance, especially when managing unpredictable intraoperative complications. In this study, we report on the surgical management of a pathological femoral neck fracture with multiple metastases with carcinoma of an unknown primary origin. Primary total hip replacement was planned. However, due to the presence of multiple metastases in the lower limb with intraoperative fracture of the distal femur, a total femoral replacement with a push-through endoprosthesis was carried out primarily. The duration for the surgical intervention was three hours and 56 minutes. Following surgery, initial intensive care was necessary due to the multimorbidity of the patient. Two days after surgery, the patient could be mobilized with full weight bearing and no restriction of range of motion of the entire left lower limb supervised by physiotherapists at ward level, which she tolerated well. The pain was significantly relieved during hospital stay. Mega-endoprostheses with push-through stems are a reliable option in cases with multiple metastases.

## Abstract (de)

Endoprothetik bei Extremitätentumoren ist anspruchsvoll. Sorgfältige Planung und die Expertise des Operationsteams sind von größter Bedeutung, insbesondere im Umgang mit unvorhersehbaren intraoperativen Komplikationen. In diesem Fall möchten wir über die operative Behandlung einer pathologischen Schenkelhalsfraktur mit multiplen Metastasen und unbekanntem Primärtumor berichten. Die Implantation einer primären Hüfttotalendoprothese war geplant. Aufgrund multipler Metastasen in der unteren Extremität mit intraoperativer Fraktur des distalen Femurs wurde primär das linke Hüftgelenk und das linke Kniegelenk mit einer kompletten Durchsteck-Endoprothese des Femurs versorgt. Der chirurgische Eingriff dauerte drei Stunden und 56 Minuten. Aufgrund der Multimorbidität der Patientin war postoperativ zunächst eine intensivmedizinische Behandlung erforderlich. Zwei Tage postoperativ konnte die Patientin unter Aufsicht von Physiotherapeuten auf Station mit Vollbelastung und ohne Bewegungseinschränkung der gesamten linken unteren Extremität mobilisiert werden, was sie gut verkraftete. Die Schmerzen ließen während des Krankenhausaufenthalts deutlich nach. Mega-Endoprothesen mit Durchsteckschäften sind bei multiplen Metastasen eine zuverlässige Option.

## Introduction

Mega-endoprostheses provide a wide range of management options for treating pathological fractures of the lower extremities [1], [2]. In this study, we report on the surgical management of a pathological femoral neck fracture with multiple metastases with carcinoma of unknown primary. Primary total hip replacement was planned. Due to multiple metastases in the lower limb with intraoperative fracture of the distal femur, total femoral replacement with push-through endoprosthesis was carried out primarily. The aim of the study is to highlight the management of intraoperative complications with decisive focus on final stability and postoperative ability to full weight bearing.

## Patient and methods

**Figure 1:**
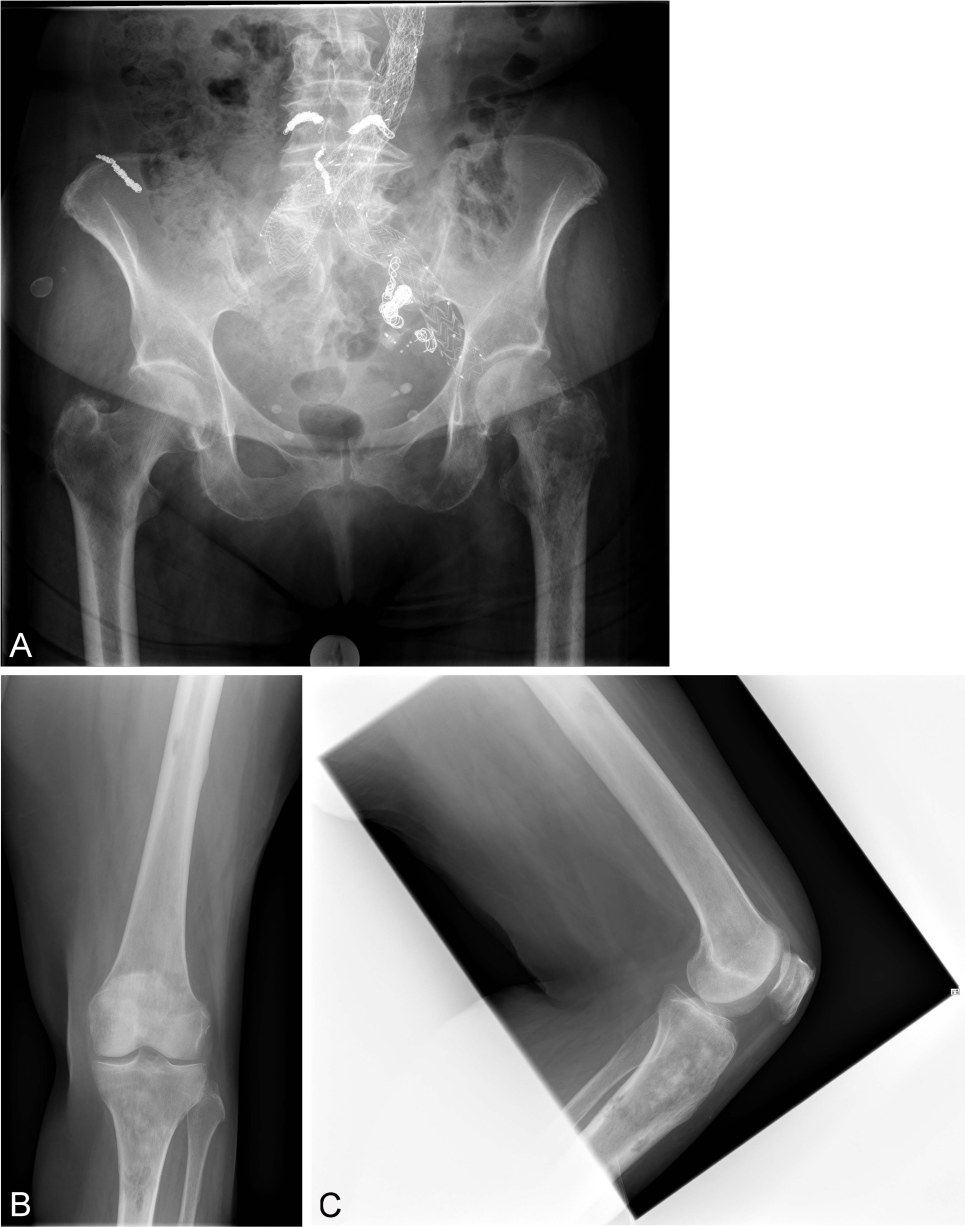
A. Preoperative X-ray of the pelvis and both hip joints: fracture of the left femoral neck with signs of infiltration of the acetabulum and of the proximal femur with metastases. B, C. Preoperative x-ray of the left knee joint, the distal femur and the proximal tibia showing signs of infiltration of the acetabulum and of the proximal femur with metastases

This is a report on a female patient aged 70 years at time of surgery. She was admitted to our center due to a pathological fracture of the left femoral neck (Figure 1A (Fig. 1)). Beside the carcinoma of unknown primary, multiple aneurysms of the aorta, cardiac insufficiency and chronic renal failure were diagnosed. Due to multiple metastases of the acetabulum as well as the whole femur (Figure 1B, C (Fig. 1)), a palliative approach involving primary total hip replacement using a long stem was planned. The oncological prognosis was not clear. 

### Surgery

We performed an anterolateral approach to the left hip joint. After preparation of the hip we implanted a cemented acetabular cup for dual mobility (BiMentum™ cemented cup, *DePuy* Synthes, 325 Paramount Drive, Raynham, MA, USA). 

**Figure 2:**
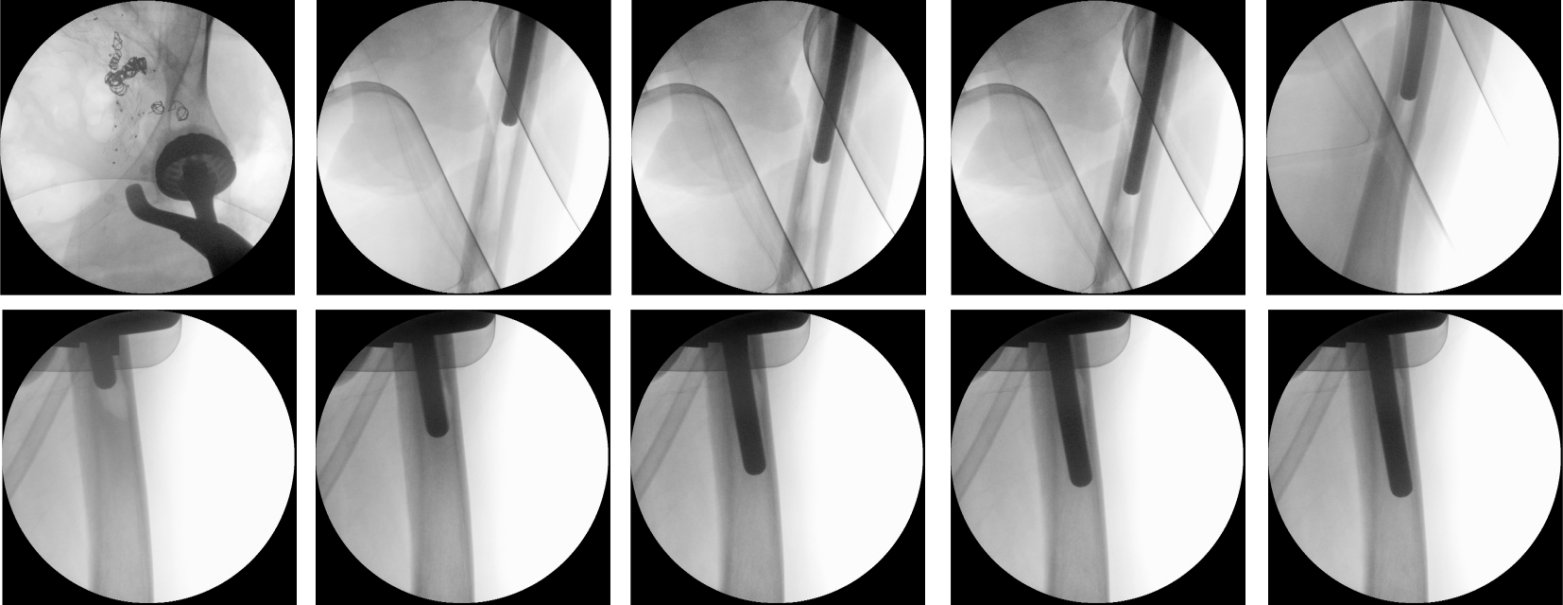
Intraoperative imaging: the femoral medullary cavity was drilled further to insert a 12 mm stem. The trial showed a very good result. Therefore, we decided to implant a 260 mm stem with a 12 mm diameter in accordance with the preoperative planning. Yet, the trial stem was stuck and could not be removed.

**Figure 3:**
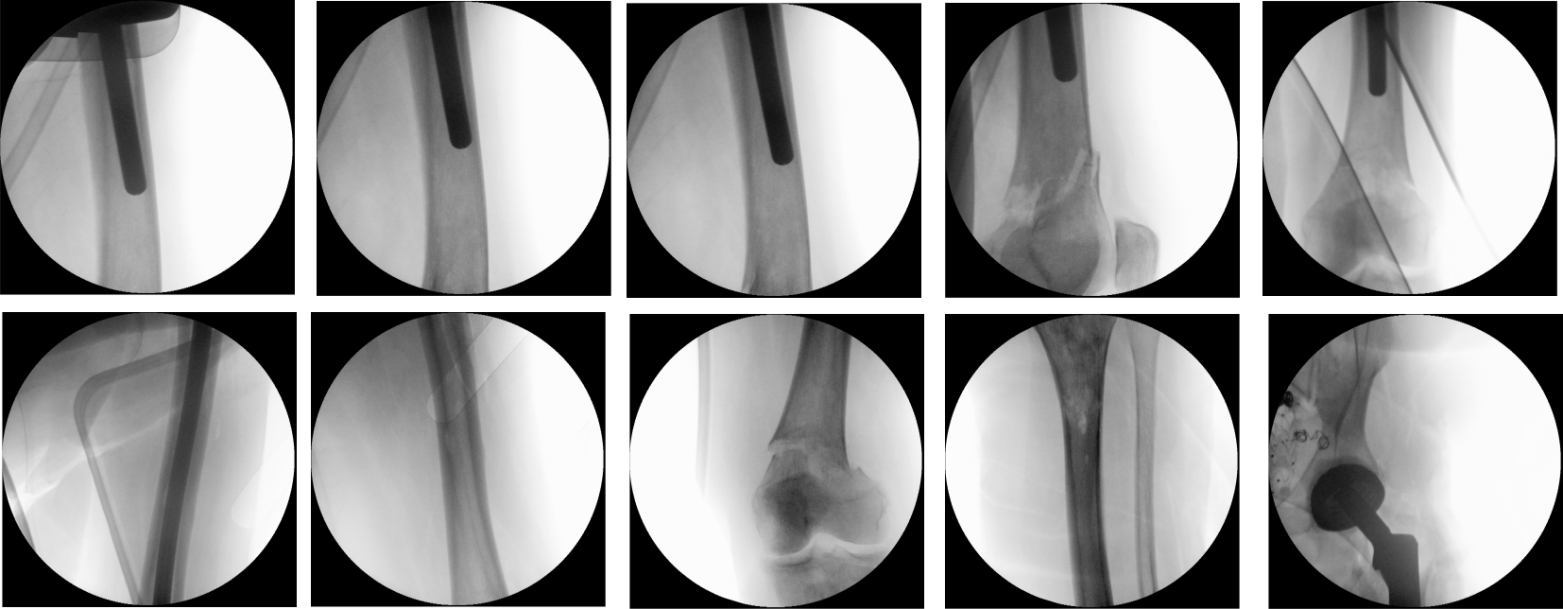
Intraoperative imaging: during repeated attempts to remove the stem, the femur fractured above the condyles. The trial stem could only be removed after splitting the femoral shaft.

**Figure 4:**
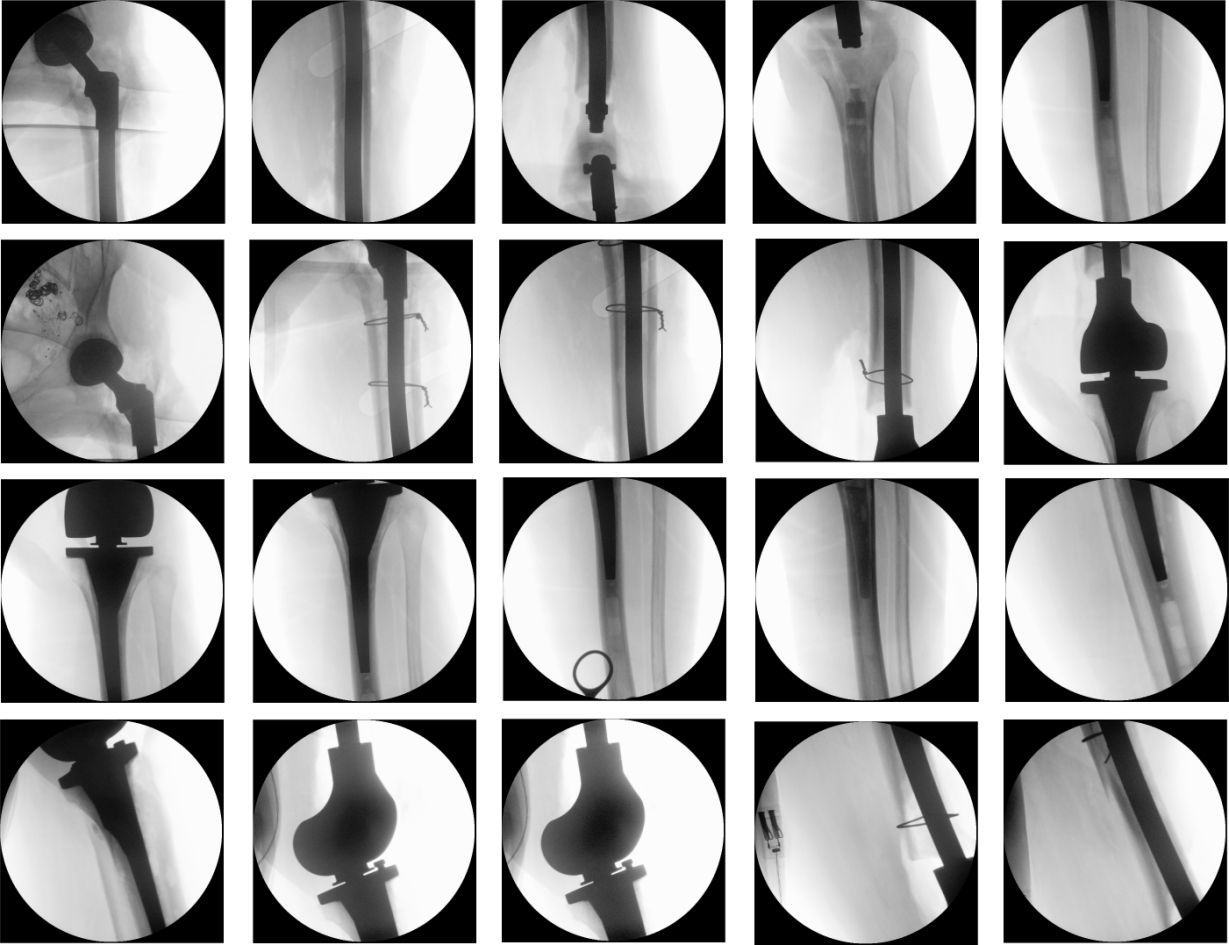
Intraoperative imaging showing the steps to replace the proximal femur and the knee joint with push-though stem of the femoral shaft

The leg was then repositioned to create access to the medullary cavity. A swab and histological specimen were also taken from the medullary cavity. The femoral medullary cavity was rasped using special rasps. The medullary cavity was narrow. The femoral shaft was drilled. The thinnest long stem of the MRP-Titan^®^ Peter Brehm (Peter Brehm GmbH, Am Mühlberg 30, 91085 Weisendorf, Germany) has a diameter of 11 mm. However, this is not suitable for patients weighing over 65 kg. Our patient, however, weighed approximately 80 kg. Since the bone structure was considerably soft and there were multiple metastases throughout the femur, the femoral medullary cavity was drilled further to insert a 12 mm stem. The trial showed a very good result (Figure 2 (Fig. 2)). Therefore, we decided to implant a 260 mm stem with a 12 mm diameter in accordance with the preoperative planning. Yet, the trial stem was stuck and could not be removed. During repeated attempts to remove it, the femur fractured above the condyles (Figure 3 (Fig. 3)). The trial stem could only be removed after splitting the femoral shaft. Definitive treatment was now being discussed. One option would have been to replace the whole femur with total hip and knee replacement using a push-through stem for the femoral shaft. The alternative would have been a Girdlestone resection arthroplasty and osteosynthesis of the distal femur with a locking plate. Because the bone structure was severely compromised by the multiple metastases, plate osteosynthesis would have been neither stable under load nor stable for exercise. For this reason, and after weighing up the advantages, risks, complications, and chances of success of the above-mentioned treatment options, we have opted for replacement of the proximal and distal femur and for a push-through endoprosthesis for adequate mobilization in a palliative treatment approach. The knee joint was accessed via a median approach. The preparation of the proximal tibia was carefully carried out, as it was full of metastases (Figure 1B, C (Fig. 1), Figure 3 (Fig. 3)). Finally, the proximal femur and the knee joint were successfully replaced with a push-through stem of the femoral shaft (Figure 4 (Fig. 4)), using the LINK^®^ Megasystem-C with the Endo-Modell SL knee component (Waldemar Link GmbH & Co. KG, Helmut D. Link, Barkhausenweg 10, 22339 Hamburg, Germany). 

## Results

**Figure 5:**
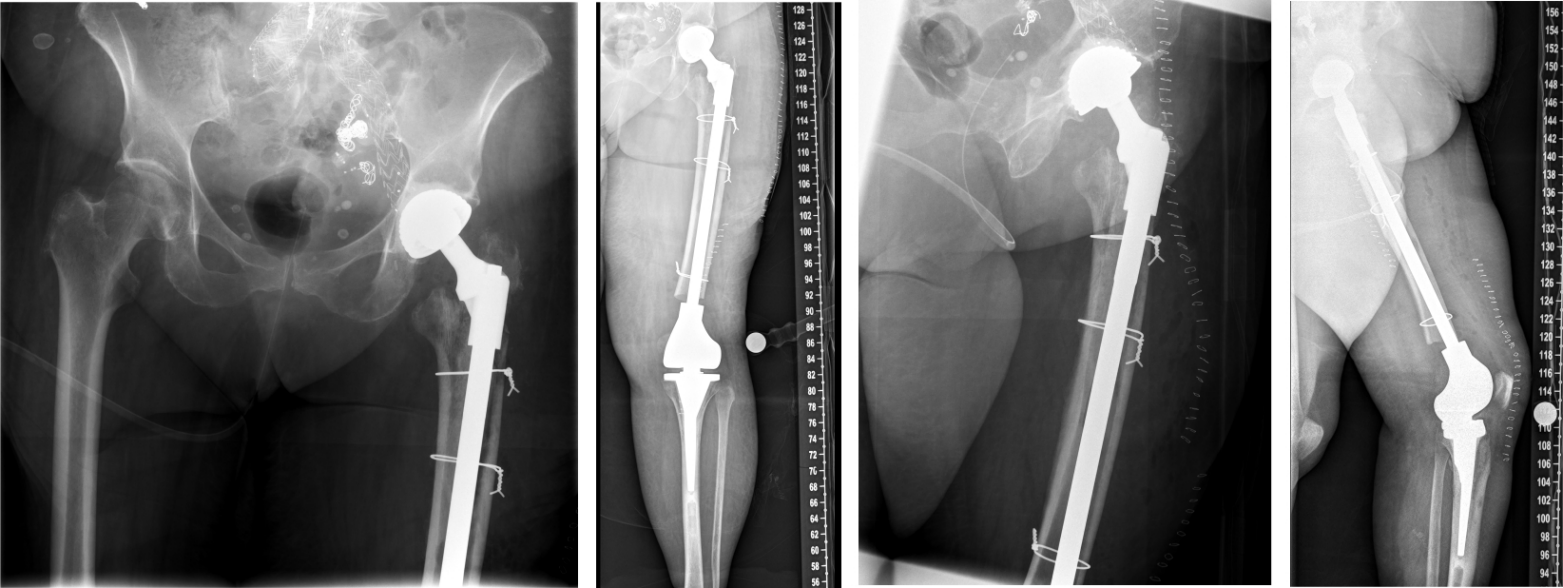
The postoperative radiographs showed correct implant position and a satisfactory surgical result.

The duration for the surgical intervention was three hours and 56 minutes. Following surgery, initial intensive care was necessary due to the multimorbidity of the patient. Two days after surgery, she could be mobilized with full weight bearing and no restriction of range of motion of the entire left lower limb supervised by physiotherapists at ward level, which she tolerated well. The pain was significantly relieved during hospital stay. The postoperative radiographs showed correct implant position and a satisfactory surgical result (Figure 5 (Fig. 5)).

Histologic examination of intraoperative material revealed angiosarcoma. The range of motion of the left hip joint two weeks after surgery was: extension/flexion 0/0/90°, abduction/adduction 30/0/20°, external rotation/internal rotation 30/0/20°. The range of motion of the left knee joint two weeks after surgery was: extension/flexion 0/0/120°. There were no symptoms or signs of infection or any other orthopaedic complications. Two weeks after surgery, the general condition of the patient deteriorated significantly. Further palliative treatment was carried out to alleviate the general and oncological condition. Yet, the general condition deteriorated significantly, with very poor prognosis. Finally, the patient died almost 4 weeks after surgery due to deteriorated general condition. 

## Discussion

After providing the patient with detailed information on the pathological fracture of the left femoral neck, and presenting the case at the staff meeting, we discussed the following treatment options with the patient:

- Conservative treatment (not medically justifiable because the patient was in pain and immobile)
- Resection arthroplasty (not favoured because we intended to adequately mobilize the patient with full weight bearing to prevent thromboembolic complications)
- Cemented total hip replacement with a standard stem (not to be favoured due to the multiple metastases throughout the left femur)
- Total hip replacement with cemented implantation of the acetabular cup and implantation of long uncemented stem with distal locking screws to stabilize the entire femur. This option was preferable to the above-mentioned methods and was attempted.

Yet, the intraoperative complication forced us to change the intraoperative strategy focusing on postoperative stability and capability of full weight bearing.

Mega-endoprostheses were first used in tumor surgery of the lower extremities [1], [2], [3], [4], [5], [6]. Most mega-implant systems provide modular components. This modularity enables considerable reconstruction options [1], [3]. However, this is major surgery with high complication rates [1], [5]. A push-through implant allows for better muscular attachment due to preservation of bone [2]. Further, it reduces the size of the cavity within the limb, which again minimizes effusion and the necessity of postoperative puncture and possible infections.

The limitation of this study lies in its retrospective design and very short follow-up period. However, the intraoperative complication and its successful management highlight the scope of arthroplasty in tumor surgery.

## Conclusion

Arthroplasty in managing tumors of the extremities is challenging. Careful planning and the expertise of the surgical team are of the utmost importance, especially in managing unpredictable intraoperative complications. Mega-endoprostheses with push-through stems are a reliable option in cases with multiple metastases.

## Notes

### Ethics approval

Approval of the local institutional review board for study had been given (Ethical Committee at the Medical Faculty, Leipzig University, AZ 020/21-ek) in view of the retrospective nature of the study, and because all the procedures were part of routine care.

### Availability of data and material

The datasets used and/or analyzed during the current study are available from the corresponding author on reasonable request.

### Informed consent

The patient has given general consent in the use of her data, including imaging data, to be used for analysis and publication. This has been approved by the Ethical Committee. 

### Author’s ORCID

Mohamed Ghanem: 0000-0003-1724-336X

### Competing interests

The authors declare that they have no competing interests.
